# Identification of biomarkers related to immune and inflammation in membranous nephropathy: comprehensive bioinformatic analysis and validation

**DOI:** 10.3389/fimmu.2023.1252347

**Published:** 2023-10-09

**Authors:** Pingna Zhang, Yunling Geng, Jingyi Tang, Zijing Cao, Xiaojun Xiang, Kezhen Yang, Hongbo Chen

**Affiliations:** ^1^ Department of Nephrology, The First Affiliated Hospital of Zhejiang Chinese Medical University (Zhejiang Provincial Hospital of Chinese Medicine), Hangzhou, China; ^2^ Renal Research Institution of Beijing University of Chinese Medicine, and Key Laboratory of Chinese Internal Medicine of Ministry of Education and Beijing, Dongzhimen Hospital Affiliated to Beijing University of Chinese Medicine, Beijing, China; ^3^ Department of Rehabilitation Medicine, Sir Run Run Shaw Hospital, Zhejiang University School of Medicine, Hangzhou, China

**Keywords:** membranous nephropathy, immune, inflammatory cytokines, biomarkers, bioinformatics

## Abstract

**Background:**

Membranous nephropathy (MN) is an autoimmune glomerular disease that is predominantly mediated by immune complex deposition and complement activation. The aim of this study was to identify key biomarkers of MN and investigate their association with immune-related mechanisms, inflammatory cytokines, chemokines and chemokine receptors (CCRs).

**Methods:**

MN cohort microarray expression data were downloaded from the GEO database. Differentially expressed genes (DEGs) in MN were identified, and hub genes were determined using a protein-protein interaction (PPI) network. The relationships between immune-related hub genes, immune cells, CCRs, and inflammatory cytokines were examined using immune infiltration analysis, gene set enrichment analysis (GSEA), and weighted gene co-expression network analysis (WGCNA). Finally, the immune-related hub genes in MN were validated using ELISA.

**Results:**

In total, 501 DEGs were identified. Enrichment analysis revealed the involvement of immune- and cytokine-related pathways in MN progression. Using WGCNA and immune infiltration analysis, 2 immune-related hub genes (*CYBB* and *CSF1R*) were identified. These genes exhibited significant correlations with a wide range of immune cells and were found to participate in B cell/T cell receptor and chemokine signaling pathways. In addition, the expressions of 2 immune-related hub genes were positively correlated with the expression of *CCR1*, *CX3CR1*, *IL1B*, *CCL4*, *TNF*, and *CCR2*.

**Conclusion:**

Our study identified *CSF1* and *CYBB* as immune-related hub genes that potentially influence the expression of CCRs and pro-inflammatory cytokines (*CCR1*, *CX3CR1*, *IL1B*, *CCL4*, *TNF*, and *CCR2*). *CSF1* and *CYBB* may be potential biomarkers for MN progression, providing a perspective for diagnostic and immunotherapeutic targets of MN.

## 1 Introduction

Membranous nephropathy (MN) is an autoimmune glomerular disease and is the most frequent cause of nephrotic syndrome (NS) in adults, accounting for approximately 30% of the cases, with individuals aged 30–50 years reaching the peak incidence rate (1, 2). Specific lesions of MN are the results of thickening of the glomerular capillary walls that results from the formation of immune deposits on the capillary wall (3). This immunological conflict is predominantly mediated by immune complex deposition and complement activation, which contribute to the impairment of glomerular filtration barrier, leading to nonselective proteinuria (4). Approximately 80% of MN cases occur without a specific cause (primary MN [pMN]), whereas 20% are associated with other diseases such as lupus erythematosus, infections (hepatitis B), malignancies, or drug intoxication (5).

The organ-specific autoimmune nature of pMN was determined in 2009 with the identification of phospholipase A2 receptor (PLA2R) in podocytes (6). Glomerular deposition of IgG on PLA2R is specific for pMN and is found in approximately 70% of cases reported in adults. This discovery has improved our understanding of the pathophysiology of pMN, in which circulating autoantibodies directly target podocyte antigens (7). This has opened a paradigm shift in the pathophysiological pattern, diagnosis, and targeted intervention for MN. Meanwhile, the mechanisms of autoimmunity initiation, exposure to antigens, and antibody pathogenicity have attracted much attention, revealing the significance of the immune system in the progression of MN (8). The initiation of MN may involve the cooperation of multiple factors, including genetic and environmental factors, as well as epigenetic and immune predispositions that result in the loss of immune system tolerance to develop MN (9). B and T cells, autoantibodies, cytokines, and complement system activation contribute to MN pathogenesis. A disordered proportion of regulatory T cells has been suggested to be the main characteristic of patients with MN without treatment (10). Some patients with MN display an increase in the CD4^+^/CD8^+^ subset ratio (11), which might be associated with the clinical response to immunosuppressive therapy; however, this was not confirmed in patients with MN treated with rituximab (12). In addition, the number of plasma cells and regulatory B cells in patients with MN was significantly higher than those in healthy individuals, and the number of PLA2R-specific memory B cells amplified *in vitro* may be related to circulating PLA2R antibody titers (13). Chemokines and chemokine receptors (CCRs) could recruit immune cells into tissues and are involved in inflammatory response (14). However, many aspects of the molecular mechanisms of immunity and chemokines involved in MN pathogenesis remain unclear.

**Figure 1:**
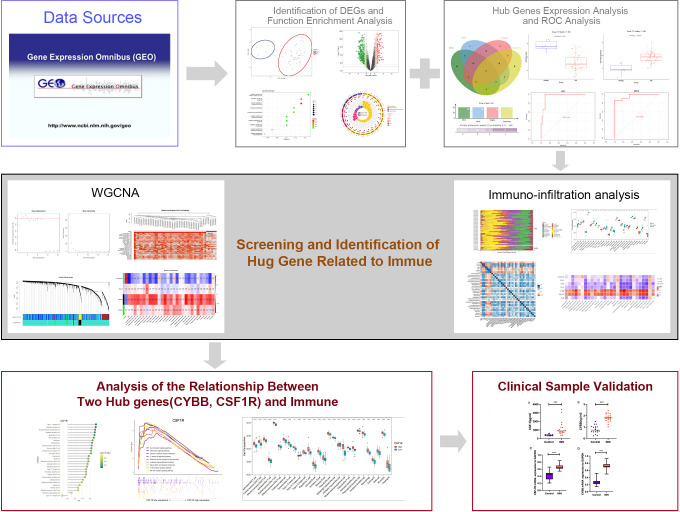
Technology roadmap for this study.

Bioinformatics analysis has been widely utilized to reveal molecular pathogenesis of diseases and identify disease biomarkers (15). Microarray technology has been used in a range of bioinformatics analyses in the biomedical field to provide novel insights and help discover critical factors in the etiopathogenesis of diseases (16). In this study, gene expression profiles of MN were obtained using the Gene Expression Omnibus (GEO) database and differentially expressed genes (DEGs) were identified from them. Notably, weighted co-expression network analysis (WGCNA) and Gene Set Enrichment Analysis (GSEA) analyses were performed to explore immune cell infiltration in MN and further reveal immune-related pathways and identify potential biomarkers for MN. In addition, correlations between immune-related central genes and pro-inflammatory cytokines were analyzed. A flowchart of this study is shown in **Figure 1**.

## 2 Materials and methods

### 2.1 Data acquirement and preprocessing

Microarray expression data for the MN cohorts were obtained from the GEO database (http://www.ncbi.nlm.nih.gov/geo/). The GSE108113 dataset containing data of 44 cases of MN and six healthy controls was used as a training set. For validation, microarray expression data from an additional 51 patients with MN and 6 healthy controls were obtained from another dataset, GSE200828. The “ggord” and “yyplot” packages in R were used to perform principal component analysis (PCA) between samples. In this analysis, the sample separation among different groups were checked. Meanwhile, the “GEOquery” package was used to perform probe annotation and normalization of gene expression using the obtained datasets. When a gene corresponded to multiple probe IDs, only the ID with the highest average expression level was retained. The standardized matrix file was used for all subsequent downstream analyses.

### 2.2 Identification of DEGs, construction of PPI network, and screening of hub genes

Differential expression analysis was conducted using the ‘limma’ package in R software, employing a screening criterion of P-value < 0.05 and |log fold change (FC)| > 1.5 (17, 18). The resulting DEGs were visualized using various approaches including PCA, Venn diagrams, and volcano plots. To identify hub genes, a protein-protein interaction (PPI) network of DEGs was constructed using the STRING database (https://cn.string-db.org/). The PPI information was extracted with an interaction score of 0.4, specifically focusing on “Homo sapiens” as the species. Subsequently, the PPI network analysis results were exported to Cytoscape 3.9.1, for further investigation. The CytoHubba plug-in was employed to sort and filter the nodes within the network based on the network characteristics, aiding in the identification of core elements within this complex network. Core genes were identified using the degree, MNC, closeness, and MCC methods available in the CytoHubba plug-in. Common genes among the top 20 genes identified by each method were identified to determine the hub genes.

### 2.3 Functional enrichment analysis

GO and KEGG analyses of the DEGs were conducted using the Metascape database (https://metascape.org/). The analysis was limited to the species “Homo sapiens,” and the KEGG screening conditions included a minimum overlap of 3, a P-value threshold of 0.01, and a minimum enrichment of 1.5. The DEG list was used for GO and KEGG enrichment analysis (19), and the results were visualized using the ‘clusterprofiler’ packages of R software and the OmicShare tools (https://www.omicshare.com/tools).

### 2.4 Validation of hub genes

The levels of different hub genes in patients with MN and healthy individuals were assessed by box plots, which were processed by the “ggplot2” package of R software. To further evaluate the predictive accuracy of the hub genes, receiver operating characteristic (ROC) curve analysis was performed to distinguish patients with MN from healthy individuals. Based on the obtained expression profile of hub genes and its high-throughput sequencing data, the ROC curves of hub genes were plotted using the “pROC “ software package. The area under the curve (AUC) was used to compare the diagnostic value of the hub genes. Meanwhile, the independent external GSE200828 dataset was used to validate the expression levels and diagnostic value of the hub genes in distinguishing patients with MN and healthy individuals.

### 2.5 Immune cell infiltration analysis and its correlation with hub genes

The ssGSEA algorithm (20) was utilized to quantify the infiltration levels of 28 immune cells in the selected samples from the GSE108113 dataset. The abundance of these 28 types of infiltrating immune cells in the GSE108113 samples was estimated using the “GSVA” package. The proportion of 22 immune cell types in the GSE108113 samples was estimated using CIBERSORTx. The relationship between hub genes and immune cell infiltration was estimated by the “GSVA” package, which was visualized using the “ggplot2” package. KEGG pathway datasets from different expression groups performed functional enrichment analyses by using “GSVA” package.

### 2.6 Weighted gene co-expression network analysis and its correlation with hub genes

To construct a gene co-expression network of the GSE108113 dataset, WGCNA was performed using the WGCNA package in R software. Genes with the highest absolute deviation of 25% from the median were selected for analysis (21). The quality of the analyzed data was evaluated by “goodSampleGenes” function, followed by clustering of samples and elimination of outlier samples. An ideal soft threshold was selected, and the “pickSoftThreshold” function was used to transform the matrix data into an adjacency matrix. This was followed by cluster analysis and modules were identified according to topological overlap. The results of immune infiltration obtained as phenotypic data were combined with WGCNA results to perform module analysis, which aimed to explore the relationship between the modules and immune cells. The genes in the module most closely related to immunity that overlapped with the hub genes were selected for further analysis.

### 2.7 Correlation of immune-related hub genes with chemokines and pro-inflammatory cytokines

To further analyze the correlation between immune-related hub genes, CCRs, and pro-inflammatory cytokines, lists of CCRs and pro-inflammatory cytokines were collected (**Supplementary Table S1**). Spearman correlation coefficients between immune-related hub genes, CCRs, and pro-inflammatory cytokines were analyzed. Thereafter, scatter plots depicting their relations were generated using the “ggstatsplot” package, presenting the linear relationship between them generated using the statistical method “lm.”.

### 2.8 Enzyme-linked immunosorbent assay

To measure the protein levels of CSF-1 and CYBB/NOX2, we used specific ELISA kits for human CSF-1 (KE00184; Proteintech, USA) and CYBB/NOX2 (EK13559; Signalway Antibody, USA). Serum samples were collected from 20 patients with MN and 18 healthy controls (the clinical characteristics of the patients with MN and healthy controls are provided in **Supplementary Table S2**). ELISA was performed according to the manufacturer’s instructions. Briefly, the standard samples and samples from both experimental groups were transferred to a 96-well plate. An equal volume of the kit reagent was added to each well, and the plate was incubated for 30 min. The stop solution was added, and the absorbance signal was measured at 450 nm using a plate reader.

### 2.9 RT-PCR

To test the gene expression level of CSF1R and CYBB in patients with MN and healthy controls, quantitative real-time polymerase chain reaction (RT–PCR) analysis of the mRNA levels of the genes from serum of patients was performed (Applied Biosystems, USA). The mRNA was reverse transcribed to cDNA using an Omniscript RT kit (Vazyme, China). RT-qPCR analysis was performed using the AceQ Universal SYBR qPCR Master Mix. After GAPDH normalization, the relative expression levels of the target gene were carried out with the 2^-△△^CT approach. The primer sequences are listed in **Table 1**.

**Table 1:** Sequences of the primers designed for RT-qPCR.

Gene	Forward sequence	Reverse sequence
CYBB	TGGAAACCCTCCTATGACTTGG	AAACCGAACCAACCTCTCACAAA
CSF1R	GCTGCTTCACCAAGGATTATG	GGGTCACTGCTAGGGATG
GAPDH	GCACCGTCAAGGCTGAGAAC	TGGTGAAGACGCCAGTGGA

### 2.10 Ethics approval and consent to participate

All patients came from Dongzhimen Hospital. Studies involving human participants were reviewed and approved by the Ethics Committee of Beijing Dongzhimen Hospital, First Clinical Medical College of Beijing University of Chinese Medicine. All the patients/participants provided written informed consent to participate in the study.

## 3 Results

### 3.1 Sorting of sample data, identification of DEGs and screening of hub genes

**Figure 2:**
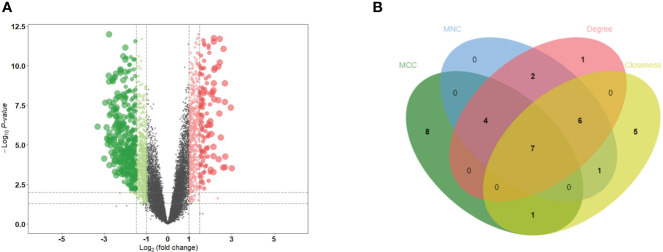
Identification of DEGs and hub genes. **(A)** Volcano diagram of DEGs. Red indicates upregulated genes and green indicates downregulated genes. **(B)** Venn diagram for screening hub genes. DEGs, differentially expressed genes.

PCA distinctly indicated the separation of patients with MN from healthy controls (**Figure 2A**). A total of 501 DEGs were identified during differential expression analysis based on adjusted P-value < 0.05 and |logFC| > 1.5. Among these DEGs, 133 were upregulated and 368 were downregulated (**Figure 1**) (**Supplementary Table S3**). Using the STRING database, a PPI network of DEGs was constructed, comprising 273 nodes and 840 edges. The scores for degree, MNC, closeness, and MCC were calculated using the CytoHubba plug-in, and the top 20 genes from each method were intersected to identify seven hub genes: *SLC2A2*, *HRG*, *CYBB*, *PCK1*, *CSF1R*, *FTCD*, and *ALB*. The Venn diagram in **Figure 2B** shows the overlap between DEGs.

### 3.2 GO and KEGG enrichment analysis of DEGs

**Figure 3:**
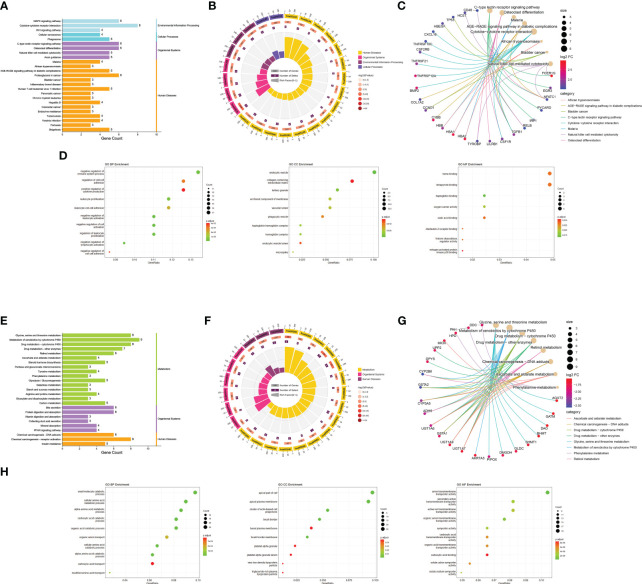
Functional enrichment analysis. **(A)** Bar graph of KEGG pathway enrichment analysis of upregulated genes. The horizontal coordinate indicates the number of genes annotated to the pathway, and different colors represent different pathway classifications. **(B)** Circle diagram of KEGG pathway enrichment analysis of upregulated genes. The first circle indicates the number of the pathway; the second circle indicates the number of genes and P-value of the pathway; the third circle indicates the number of genes annotated to the pathway; and the fourth circle indicates the enrichment coefficient for each pathway. **(C)** Network diagram of KEGG pathway enrichment analysis of upregulated genes indicate the specific target distribution on the pathway. **(D)** Bubble plot of GO enrichment analysis of upregulated genes, including BP, CC, and MF. The size of the dots indicates the number of genes, and the color corresponds to the P-value. **(E-H)** GO and KEGG pathway enrichment analysis of downregulated genes. BP: biological process, CC: cell component, MF: molecular function.

During KEGG enrichment analysis, the upregulated KEGG pathways were related to the MAPK signaling pathway, cytokine-cytokine receptor interactions, and NK cell-mediated cytotoxicity (**Figures 3A-C**). During GO analysis, the upregulated genes were mainly involved in the negative regulation of the immune system process, endocytic vesicles, and heme binding (**Figure 3D**). Among the downregulated genes, the most relevant downregulated KEGG pathways were those related to metabolism of xenobiotics by cytochrome P450, drug metabolism, and chemical carcinogenesis-receptor activation (**Figures 3E-G**). The genes downregulated in MN were mostly related to amino acid metabolic process, apical part of cell, and transmembrane transporter activity (**Figure 3H**). Detailed information is provided in **Supplementary Table S4**.

### 3.3 Validation of hub gene expression and diagnostic efficacy

**Figure 4:**
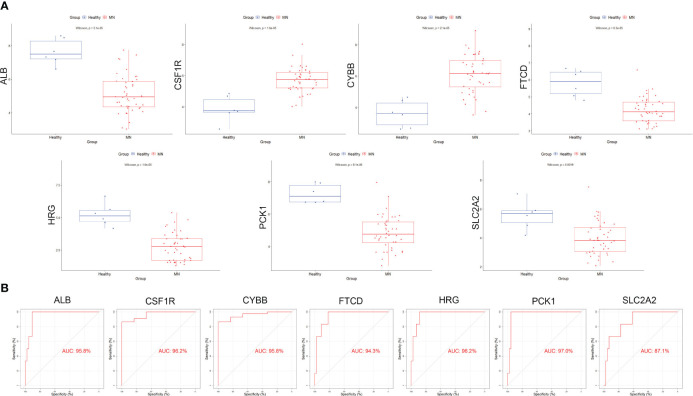
Validation of hub genes in the exploration cohort. **(A)** The expression of each hub gene in different subgroups. **(B)** AUC values for each hub gene in the ROC curve. AUC: area under the curve. ROC: receiver operating characteristic.

**Figure 5:**
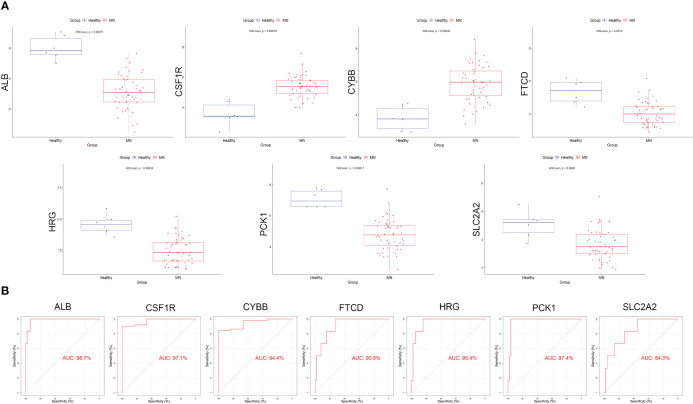
Validation of the hub genes in the validation cohort. **(A)** The expression of each hub gene in different subgroups. **(B)** AUC values for each hub gene in the ROC curve.

Box plots were constructed to assess the expression levels of the seven hub genes between patients with MN and healthy controls (**Figure 4A**). The expression levels of *ALB* (P=2.1e-05), *FTCD* (P=6.3e-05), *HRG* (P=1.6e-05), *PCK1* (P=8.1e-06), and *SLC2A2* (P=0.0019) in MN were significantly lower than those in healthy controls. However, the expression levels of *CSF1R* (P=1.6e-05) and *CYBB* (P=2.1e-05) in patients with MN were significantly higher than those in healthy controls (**Figure 4B**). Furthermore, to validate the expression levels of these seven hub genes in patients with MN and healthy controls, an independent external dataset, GSE200828, was used, and the results were consistent with those from the GSE108113 dataset (**Figure 5A**). The diagnostic ability of these seven hub genes was validated using GSE200828 dataset. The values of AUC of the seven hub genes showed that all seven hub genes indicated favorable diagnostic value for MN, with an AUC of 0.958 (95%CI 90.52%-100%) for *ALB*, AUC of 0.962 (95%CI 90.53%-100%) for *CSF1R*, AUC of 0.958 (95%CI 90.23%-100%) for *CYBB*, AUC of 0.943 (95%CI 87.22%-100%) for *FTCD*, AUC of 0.962 (95%CI 91.1%-100%) for *HRG*, AUC of 0.970 (95%CI 92.44%-100%) for *PCK1*, and AUC of 0.871 (95%CI 73.74%-100%) for *SLC2A2* (**Figure 5B**). Thus, all seven hub genes exhibited high diagnostic values, with AUC values > 0.85.

### 3.4 Immune cell infiltration and association between hub genes

**Figure 6:**
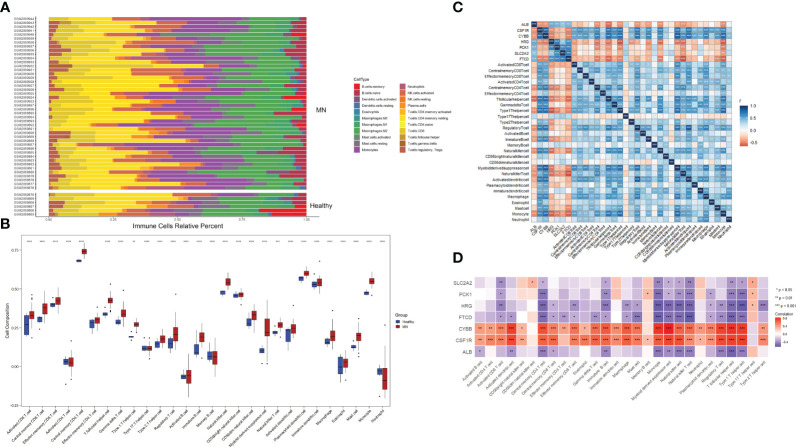
Immune cell infiltration analysis. **(A)** Plot of the percentage of 22 immune cells in each sample. Different colors represent different immune cells. **(B)** The distribution of 28 immune cells in different groups. **(C, D)** Correlation between 28 immune cells and hub genes. The color indicates the correlation size. *P <0.05, **P <0.01, ***P <0.001, ****P <0.0001.

To investigate the relative level of immune cell infiltration between patients with MN and healthy controls, the CIBERSORT algorithm was used. The distribution of the 22 immune cell infiltrations in GSE108113 is presented as a bar plot (**Figure 6A**). Immune cell infiltration analysis revealed that the numbers of CD4^+^ T cells, CD8^+^ T cells, natural killer (NK) cells, monocytes, and macrophages were significantly higher in MN tissues than in healthy tissues (*P <* 0.001) (**Figure 6B**). Furthermore, the correlation between the seven hub genes and 28 immune cells was assessed. *CYBB* and *CSF1R* exhibited positive correlations with several immune cells, particularly central memory CD4^+^ T cells (cor = 0.70, *P <* 0.001; cor = 0.640, *P <* 0.001), monocyte (cor = 0.776, *P <* 0.001; cor = −0.640, *P <* 0.001), activated dendritic cell (cor = 0.750, *P <* 0.001; cor = 0.724, *P <* 0.001), T follicular helper cell (cor = 0.782, *P <* 0.001; cor = 0.744, *P <* 0.001) and regulatory T cell (cor = 0.737, *P <* 0.001; cor = 0.715, *P <* 0.001). In addition, *SLC2A2, PCK1, HRG, FTCD*, and *ALB* levels were negatively correlated with most immune cells, including central memory CD4^+^ T cells, monocytes, NK cells, and follicular helper T cells (*P <* 0.05) (**Figures 6C, D**).

### 3.5 Co-expression network construction and hub module identification

**Figure 7:**
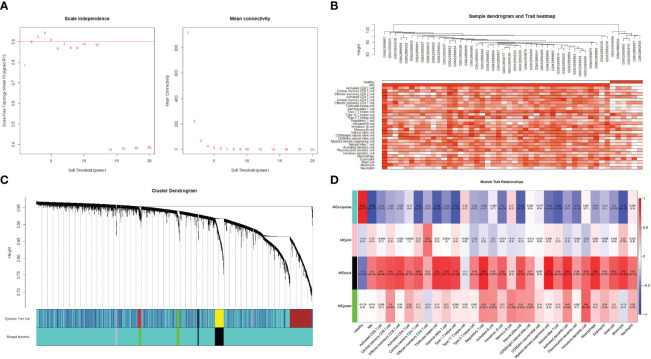
Weighted gene co-expression network analysis **(A)** Determination of the soft threshold value. The red line indicates the soft threshold value corresponding to a correlation coefficient of 0.9. **(B)** The samples were analyzed for clustering, observed for the presence of outliers, and combined with the results of immune cell infiltration. **(C)** Clustered dendrogram of the top 25% absolute deviations of the median, with each branch representing one gene. Each color at the bottom indicates one module, and the modules after merging are shown below. **(D)** Heat map of the relationship between modules and traits. The black modules in the graph clearly correlate more strongly with multiple immune cells than other modules.

For WGCNA, 6,221 genes were selected. The appropriate soft threshold was determined to be β = 2, indicating a scale-free network (**Figure 7A**). Combining the results of immune infiltration with WGCNA, the correlation between each sample and the 28 immune cells is shown in **Figure 7B**. Four gene modules were obtained by merging similar modules (**Figure 7C**). As revealed by heat maps, the correlations between multiple modules of 28 immune cells associated with patients with MN and healthy controls are presented in **Figure 7D**, which shows that immune cells are closely associated with genes in the black module. Within the black module, two immune-related hub genes, *CSF1R* and *CYBB*, were selected by intersecting the genes in the module with previously identified hub genes.

### 3.6 Correlation analysis between two hub genes and immune cell infiltration

**Figure 8:**
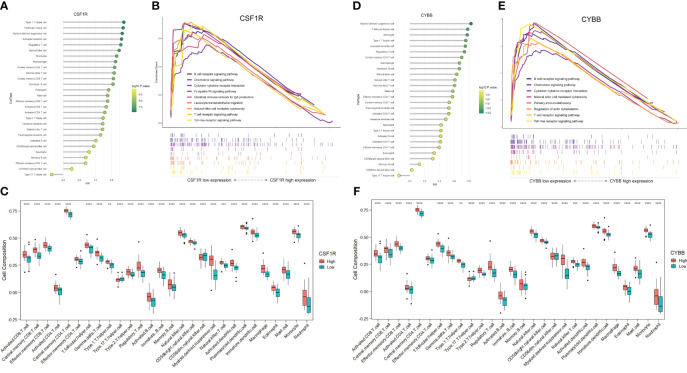
Analysis of immune-associated hub genes. **(A)** Lollipop plot of CSF1R correlation with 28 immune cells. The horizontal coordinate indicates the correlation size, and the color indicates the P-value. **(B)** Immune-related GSEA analysis of CSF1R with different expression levels. **(C)** Distribution of 28 immune cells in CSF1R with different expression levels. **(D-F)** Analysis of CYBB. **P <0.01, ***P <0.001, ****P <0.0001.

To further explore the correlation between two hub genes and immune cells infiltration, the “GSVA” and “ggplot” packages were used for analysis and visualization. As shown in **Figures 8A, D**, both *CSF1* and *CYBB* displayed a significant positive correlation with 28 immune cells, except type 17 helper cells (*P <*0.005). In **Figures 8C, F**, the boxplot shows the abundance of 28 immune cells corresponding to hub genes at different expression levels. Due to the similarity in the grouping of these two genes at different expression levels, the results of immune cell infiltration analysis are also similar, but they are actually different. CD4^+^ T cells, CD8^+^ T cells, NK cells, monocytes, and macrophages evidently exhibited high immune scores for high *CYBB* and *CSF1R* expression (*P <* 0.001), which further verified that the two hub genes were responsible for the pathogenesis of immune-mediated MN.

The GSEA of DEGs that take the canonical pathways gene sets (c2.cp.kegg.v7.5.1.symbols) in the MsigDB database as a reference was performed with the criteria of | normalized enriched score (NES)| > 1 and FDR < 0.25 (**Supplementary Table S5**). The samples were divided into high- and low-expression groups based on the expression levels of *CYBB* and *CSF1R* (**Figures 8B, E**). Overexpression of *CSF1R* was enriched in pathways involved in B cell/T cell receptor signaling pathways, chemokine signaling pathway, NK cell-mediated cytotoxicity, and cytokine-cytokine receptor interactions (*P <* 0.05). In addition, the pathways altered by *CYBB* were related to B cell/T cell receptor signaling pathways, chemokine signaling pathway, and primary immunodeficiency (*P <* 0.05). These results confirmed that *CYBB* and *CSF1R* play crucial roles in immune-related signaling pathways during MN development.

### 3.7 Correlation analysis of immune-related hub genes with ccrs and pro-inflammatory cytokines

**Figure 9:**
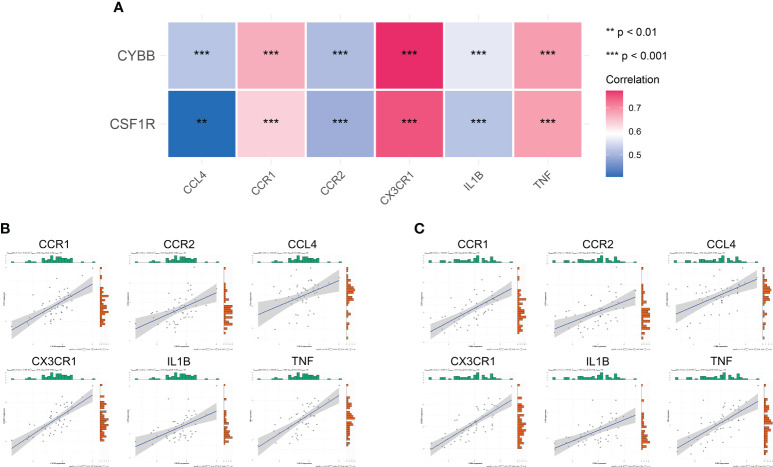
Correlation between immune-related hub genes and inflammatory factors and CCRs. **(A)** Correlation heat map. The different colors represent the magnitude of the correlation. **(B, C)** Correlation scatter plot. **P < 0.01, ***P < 0.001.

To further analyze the effect of immune-related hub genes on CCRs and pro-inflammatory cytokines, six CCRs and pro-inflammatory cytokines were obtained from the black modules: *CCR1*, *CX3CR1*, *IL1B*, *CCL4*, *TNF*, and *CCR2*. The correlation between *CYBB* and *CSF1R* and the six CCRs and pro-inflammatory cytokines is shown in **Figure 9A**. There was a significant positive correlation with a significant difference (*P <* 0.01). The correlation between *CSF1R* and *CYBB* was further demonstrated using scatter plots (**Figures 9B, C**). As the expression of *CYBB* and *CSF1R* increased, the expression of the six CCRs and proinflammatory cytokines also increased. This showed that *CYBB* and *CSF1R* influenced the expression of CCRs and pro-inflammatory cytokines to promote inflammation.

### 3.8 Validation of CSF-1 and CYBB in clinical samples

**Figure 10:**
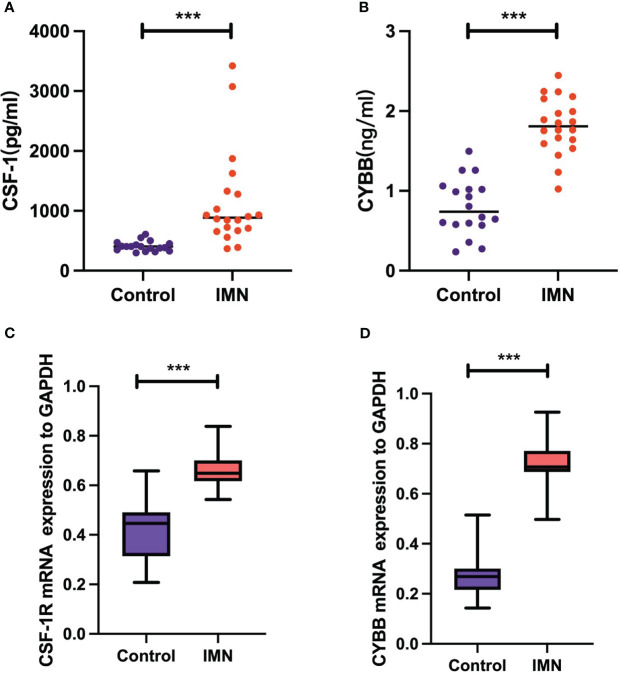
Validation of CSF-1 and CYBB. Each point represents a sample. **(A, B)** ELISA measurement of CSF-1 and CYBB. **(C, D)** mRNA expression of CSF1R and CYBB was analyzed by RT-qPCR. ***P < 0.001, vs Control.

The experimental results of serum levels of *CSF-1* in patients with MN were significantly higher than those in healthy controls (**Figure 10A**), which is consistent with the results of our bioinformatic prediction. Similarly, increased *CYBB* expression was observed in patients with MN compared to that in healthy controls (**Figure 10B**). qPCR analysis showed that mRNA expression of *CSF1R* and *CYBB* was increased in patients with MN compared to healthy controls (**Figures 10C, D**). These experimental findings validate and reinforce the predictive value of bioinformatic analysis, further supporting the involvement of *CSF1R* and *CYBB* in MN pathogenesis.

## 4 Discussion

In this study, GO analysis revealed that the upregulated genes were primarily enriched in immune system processes, endocytic vesicles, and heme-binding. The pathogenesis of MN involves the formation of circulating immune network complexes and activation of autoreactive immune cells targeting glomerular cells, encompassing both innate and adaptive immune responses. The upregulated genes mainly participated in multiple immune-related diseases and immune pathways according to KEGG, such as the C-type lectin (CTL) receptor (CLR) signaling pathway, cytokine-cytokine receptor interaction, and NK cell-mediated cytotoxicity. The accumulation of subepithelial immunocomplexes induces complement activation and disruption events that lead to the release of pathogen-associated molecular patterns (PAMP) and damage-associated molecular patterns (DAMP). CTLs are pattern recognition receptors that recognize these molecules and play vital roles in the immune system. CLRs on the surface of dendritic cells can promote the expression of costimulatory molecules and enhance their ability to present antigens to CD4^+^ and CD8^+^ T cells after binding to ligands, thus regulating the adaptive immune response. After recognizing ligands, CLRs on the surface of lymphocytes can induce the expression of pro-inflammatory factors and facilitate the binding of lymphocytes to major histocompatibility complex I (MHC I) or MHC class I molecules on target cells to induce inflammation and cytotoxicity. The concordance between these findings and the enrichment of upregulated genes suggests the presence of key genes involved in the immune response networks associated with MN.

Growing evidence regarding the pathogenic role of the impaired immune system and the molecular mechanism of MN progression has been widely revealed. Autoantibodies, immune complexes, and cytokines induced by T cells, B cells, monocytes, and other immune cells are involved in the progression of MN. Immune complex deposition within the GBM leads to the release of DAMP, which activate innate immune cells and trigger antigen-presenting cell (APC) activation. Antigen presentation by APCs to T cells leads to their activation and differentiation into subsets, such as Th1 and Th2, which involve key signaling pathways in patients with idiopathic MN (8). T cell-derived cytotoxicity contributes to the inflammatory response and tissue infiltration by immune cells (22). Autoreactive T-cells recruit, activate, and proliferate B-cells, resulting in the production of autoantibodies that cause barrier disruption and irreversible kidney damage. Indeed, basic and clinical research has indicated that the deposition of IgG along the glomerular basement membrane secreted by B cells is a hallmark of MN, which results in a sequence of events that impair the glomerular filtering barrier and induce proteinuria (23). In addition, B cells are present in renal biopsy specimens of MN, which shows that B lymphocytes are related to the pathogenesis of the disease (24). Rituximab treatment reduces glomerular IgG4 and C3 deposition by suppressing autoantibody generation, improving proteinuria in MN (25). Monocytes and macrophages are critical drivers of the innate immune system and are responsible for tissue regeneration and regulation immune (26). A recent study showed that CD14^+^CD163^+^CD206^+^M2 monocytes positively correlated with 24 h urine albumin and PLA2R levels in MN (27). Tubulointerstitial injury is mediated by macrophage migration, which is a common finding during the early phase of MN progression. M2-like monocytes are considered potential indicators of MN severity (28). We employed computational approaches such as CIBERSORTx or the ssGSEA algorithm to analyze immune infiltration. The results of the present study are consistent with previous findings, confirming an altered distribution of immune cells in MN.

Based on the genes closely associated with MN, as identified by PPI network analysis, seven hub genes were identified, namely, *ALB, FTCD, HRG, PCK1*, and *SLC2A2* (upregulated genes) and *CSF1R* and *CYBB* (downregulated genes). To identify genes closely related to MN progression, WGCNA was used to identify core molecules. Furthermore, combined with immune cell infiltration analysis, the results of WGCNA revealed that the black module was closely related to immune response in MN, which was further intersected with seven hub genes to obtain the overlapping genes *CSF1R* and *CYBB*. These results strongly suggest that the regulation of *CSF1R* and *CYBB* may influence MN pathogenesis through the immune system. Based on the immune infiltration analysis, both *CSF1R* and *CYBB* were significantly and positively associated with most immune cells, which further confirmed that these two genes play a central role in the autoimmune pathology of MN.

The identification of the roles of *CSF1R* and *CYBB* in MN advances our strategies for disease diagnosis and treatment. As a central receptor on the macrophage surface, *CSF1R* binds to CSF-1 or IL-34 to regulate the development, activation, and function of macrophages (29). Furthermore, the CSF-1R signaling pathway is responsible for migration and multiply of macrophage (30). Several studies have identified CSF-1R as a pharmacological target for alleviating disease progression, including those of rheumatoid arthritis, Alzheimer’s disease, and cancer. Particularly, CSF-1 acted as a “master switch” and contributed to monocyte and macrophage phenotypes that was positively related with lupus activity in kidney diseases (31). Further, the treatment with CSF-1R inhibitor was confirmed to significantly ameliorate renal injury in murine lupus (32). Pharmacological inhibition of CSF-1R with GW2580 alleviated ischemia-induced renal injury by reducing M2 macrophage infiltration (33). Recently, MALDI–MSI analysis was performed to detect proteomic alterations in renal biopsies, and macrophage migration inhibitory factor was identified as a valuable biomarker for response to therapy in MN (34). In addition, renal biopsies from patients with MN showed that monocytes/macrophages predominate the interstitial infiltrate, suggesting that macrophages may be key regulators of the pathogenesis of MN (35). Based on these studies, *CSF1R* was predicted to contribute to the disturbance of the immune balance associated with MN. *CYBB* (also called NOX2) is considered the central component of NADPH oxidase, which is responsible for the bactericidal activity within macrophages and neutrophils involved in respiratory bursts (36). When CYBB/NOX2, the terminal component of the respiratory chain, is activated, it enters the plasma membrane to form phagosomes, which are necessary for triggering superoxide production activity of the complex (37). A recent study showed that CYBB/NOX2 in cDCs promotes antigen presentation to activate CD4^+^ T cells and leads to TH cell-induced tissue damage (38). In a model of hyperhomocysteinemia-induced renal injury, NADPH oxidase-mediated redox signaling was responsible for switching on NLRP3 inflammasome activation, which recruited immune cell infiltration, ultimately leading to glomerular injury and sclerosis (39). These results are further supported by our study. To explore the possible mechanism by which *CSF1* and *CYBB* act on immune cells in MN, GSEA was used to determine the immune function of DEGs. The results revealed that *CSF1R* and *CYBB* were significantly correlated with B cell/T cell receptor signaling pathways, which are involved in MN immunopathogenesis. Furthermore, using ELISA, CSF1 and CYBB/NOX2 were found to be overexpressed in patients with MN, and the dependability of their diagnostic values was confirmed by ROC curve analysis, which further verified our bioinformatics analysis results.

Chemokines could promote differentiation of immune cells and induce tissue extravasation (40). Notably, studies have found that podocytes can be stimulated by the inflammatory setting of glomerulonephritis, which is mediated by CCRs (41). The expression of IL-10 and CCR1 mRNA were observed in polarized Mφ, and M-CSF restored the synthesis of IL-10 through M1 Mφ (42). CCL2/MCP-1 coordinates inflammatory monocyte transport between bone marrow, circulation and atherosclerotic plaque by binding to CCR2 (43).The distribution pattern of CX3CR1 was consistent with the expression of T cells and monocytes/macrophages, and it was distributed in both renal interstitial and glomerular infiltrated leukocytes (44). In our study, CCR1, CX3CR1, IL1B, CCL4, TNF, and CCR2 were activated by CSF1 and CYBB/NOX2, which are closely related to the inflammatory response. These results reveal that CSF1 and CYBB/NOX2 could be potential diagnostic biomarkers and immunotherapy targets for MN.

Our study has some limitations. First, the sample size of healthy controls was small, which may have influenced the study results. Second, through validation using sera from patients with MN, gene expression levels in renal tissues need to be further explored, and experimental studies are warranted to utilize our results in clinical settings. Third, there was little clinical information in the included dataset, which resulted in further correlation analyses that could not be conducted.

## 5 Conclusion

Through comprehensive bioinformatics analysis, we identified two hub genes (*CSF1R* and *CYBB*) that are closely involved in the progression of MN. The results of this preliminary study highlight the significance of immune infiltration and the relationship between the two hub genes and most immune cells with potential immune mechanisms in MN. *CSF1R* and *CYBB* influenced the expression of CCRs and proinflammatory cytokines (*CCR1*, *CX3CR1*, *IL1B*, *CCL4*, *TNF*, and *CCR2*). *CSF1R* and *CYBB* may be potential biomarkers for MN progression, providing a perspective for diagnostic and immunotherapeutic targets of MN.

## Data availability statement

The original contributions presented in the study are included in the article/**Supplementary Material**. Further inquiries can be directed to the corresponding authors.

## Ethics statement

The studies involving humans were approved by the Ethics Committee of Beijing Dongzhimen Hospital, First Clinical Medical College of Beijing University of Chinese Medicine. All the patients/participants provided written informed consent to participate in the study. The studies were conducted in accordance with the local legislation and institutional requirements. The participants provided their written informed consent to participate in this study.

## Author contributions

PZ: Writing – original draft. YG: Data curation. JT: Formal analysis. ZC: Software. XX: Validation. KY: Methodology. HC: Project administration. All authors contributed to the article and approved the submitted version.
